# Cohort study of patients with advanced cancer: outcomes associated with duration of antibiotic therapy for non-ventilator hospital-acquired pneumonia

**DOI:** 10.1017/ash.2024.54

**Published:** 2024-04-18

**Authors:** Seohyuk Lee, Jemma Benson, Avi Cohen, Vincent Quagliarello, Manisha Juthani-Mehta, Rupak Datta

**Affiliations:** 1 Department of Medicine, Beth Israel Deaconess Medical Center, Boston, MA, USA; 2 Section of Infectious Diseases, Yale School of Medicine, New Haven, CT, USA; 3 Section of Infectious Diseases, Boston Medical Center, Boston, MA, USA; 4 Department of Epidemiology of Microbial Diseases, Yale School of Public Health, New Haven, CT, USA; 5 Hospital Epidemiology and Infection Prevention Program, Veterans Affairs Connecticut Healthcare System, West Haven, CT, USA

## Abstract

In this single-center observational study of 118 older adults with advanced cancer who developed non-ventilator hospital-acquired pneumonia, prolonged antibiotic durations (8–14 and ≥15 vs ≤7 d) were not associated with reduced adjusted odds of 90-day all-cause readmission or death. These data may inform antimicrobial stewardship efforts in palliative care settings.

## Background

In the United States, many older adults live with advanced cancer, for whom the primary goal of care is symptom alleviation.^1^ These patients are vulnerable to hospitalization and infection due to a confluence of host- and treatment-associated factors.^2^ Hospital-acquired pneumonia is the most common and morbid healthcare-associated infection.^3^ A recent systematic review showed comparable efficacy between short and long durations of therapy for hospital-acquired pneumonia.^4^ However, prior studies have not evaluated outcomes among older adults with advanced cancer, particularly with respect to non-ventilator hospital-acquired pneumonia (NV-HAP). To inform antimicrobial stewardship efforts, we evaluated the association between the duration of therapy for NV-HAP and clinical outcomes among older adults who received palliative chemotherapy for advanced cancer.

## Methods

We studied a cohort of patients 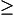 65 years of age with advanced cancer who had developed NV-HAP after receipt of palliative chemotherapy at Yale New Haven Hospital, a 1,541-bed tertiary care center in New Haven, Connecticut, from January 1, 2016, through September 30, 2017. Advanced cancer was defined as stage III–IV solid tumors and acute, refractory, and relapsed or active liquid tumors requiring chemotherapy or targeted therapies including lymphoma, leukemia, and multiple myeloma. Advanced cancers were identified by the *International Classification of Diseases (ICD)*, *Tenth Revision* codes and confirmed via pathology or medical record review. We defined cases of NV-HAP using Centers for Disease Control and Prevention National Healthcare Safety Network criteria for clinically defined pneumonia, restricted to the index event following receipt of palliative chemotherapy.^5^ We excluded patients presenting with complicated pneumonias associated with abscess, bacteremia, or fungal, necrotizing, or organizing pneumonia due to the expected extended duration of antibiotic therapy. We also excluded patients readmitted or transferred from another institution for any reason due to potential confounding with all-cause readmissions and mortality within 90 days of discharge. The Yale Human Investigation Committee approved this study.

For each patient, we collected demographics, comorbidities, history of multidrug-resistant organisms, and hospitalization characteristics including intensive care unit admission for any period of time. We also evaluated inpatient microbiological tests. All antibiotics administered orally, intravenously, or intramuscularly for NV-HAP were recorded from pharmacy data and confirmed by medical record review. For antibiotics, we determined the total days of therapy, including both inpatient and postdischarge days of therapy. We also evaluated the antibiotic spectrum index, a metric designed to compare a spectrum of activity ranging from the most narrow at 1 (eg, dicloxacillin) to the broadest spectrum at 13 (eg, tigecycline). We recorded the total antibiotic spectrum index per patient per day.^6^

We evaluated the distribution of days of therapy and determined all-cause readmissions, all-cause mortality, detection of *Clostridioides difficile*, and detection of a multidrug-resistant organism within 90 days of discharge for all patients. A multidrug-resistant organism was defined as methicillin-resistant *Staphylococcus aureus*, vancomycin-resistant enterococci, extended-spectrum β-lactamase-producing Enterobacteriaceae, or carbapenem-resistant Enterobacteriaceae. To evaluate the association between antibiotic exposure and readmission or death, we used a multivariable logistic regression model adjusted for duration of therapy (≤7 d vs 8–14 d and ≥15 d) and intensive care unit admission. We calculated adjusted odds ratios and 95% CI. We used R version 3.6.0 software (Vienna, Austria) for analyses.

## Results

### Patient characteristics

We identified 152 older adults with advanced cancer who developed NV-HAP following receipt of palliative chemotherapy. Of these, we excluded 11 due to complicated pneumonia and 23 due to readmission (n = 22) or transfer from another facility (n = 1) for any reason. Table 1 describes the remaining 118 patients included in analyses. Overall, the median age was 75 years (interquartile range, 70–81), 36.4% were female, and 28.0% had liquid tumors.

**Table 1.** Characteristics of study cohort stratified by days of antibiotic therapy (n = 118)

**Characteristic** ^**a**^	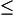 **7 d (n = 20)**	8–14 d (n = 76)	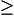 **15 d (n = 22)**
Age, years			
65–74	7 (35.0)	33 (43.4)	11 (50.0)
75–84	13 (65.0)	29 (38.2)	10 (45.5)
85+	0 (0)	14 (18.4)	1 (4.5)
Sex			
Male	15 (75.0)	50 (65.8)	10 (45.5)
Female	5 (25.0)	26 (34.2)	12 (54.5)
Race			
White	16 (80.0)	67 (88.2)	17 (77.3)
Black	2 (10.0)	6 (7.9)	4 (18.2)
Other	2 (10.0)	3 (3.9)	1 (4.5)
Ethnicity			
Hispanic	2 (10.0)	3 (3.9)	1 (4.5)
Non-Hispanic	18 (90.0)	73 (96.1)	21 (95.5)
Cancer type			
Liquid tumor	2 (10.0)	22 (28.9)	9 (40.9)
Leukemia/lymphoma	2 (10.0)	13 (17.1)	4 (18.2)
Multiple myeloma	0 (0)	9 (11.8)	5 (22.7)
Solid tumor	18 (90.0)	54 (71.1)	13 (59.1)
Gastrointestinal	4 (20.0)	13 (17.1)	2 (9.1)
Genitourinary	2 (10.0)	14 (18.4)	1 (4.5)
Lung	10 (50.0)	20 (26.3)	5 (22.7)
Other	2 (10.0)	7 (9.2)	5 (22.7)
Comorbidities			
Chronic cardiac disease	19 (95.0)	69 (90.8)	22 (100)
Chronic renal disease	0 (0)	11 (14.5)	3 (13.6)
Chronic pulmonary disease	8 (40.0)	29 (38.2)	10 (45.5)
Chronic hepatic disease	1 (5.0)	4 (5.3)	0 (0)
Neurologic disease	3 (15.0)	12 (15.8)	4 (18.2)
Prior history of MDRO			
MRSA	5 (25.0)	4 (5.3)	1 (4.5)
VRE	3 (15.0)	10 (13.2)	1 (4.5)
ESBL	0 (0)	1 (1.3)	0 (0)
CRE	0 (0)	3 (3.9)	0 (0)
Hospital characteristics			
Length of stay, days, median (IQR)	10 (8–13)	9 (8–12)	15 (14–21)
Intensive care unit^b^ admission	6 (30.0)	13 (17.1)	6 (27.3)
Intubation	1 (5.0)	7 (9.2)	4 (18.2)
Antibiotic spectrum index^c^			
Total daily score, median (IQR)	18 (12–22)	22 (18–27)	26 (20–30)
90-day clinical outcomes			
*C. difficile* detection	2 (40.0)	3 (17.6)	1 (12.5)
New MDRO detection	1 (6.3)	8 (12.1)	2 (10.5)
All-cause readmission	10 (50.0)	32 (42.1)	9 (40.9)
All-cause mortality	8 (40.0)	20 (26.7)	11 (50.0)

Note. Abbreviations: MRSA, methicillin-resistant *Staphylococcus aureus*; VRE, vancomycin-resistant enterococci; ESBL, extended-spectrum β-lactamase-producing Enterobacteriaceae; CRE, carbapenem-resistant Enterobacteriaceae; IQR, interquartile range; MDRO, multidrug-resistant organism.

a All characteristics are presented as number (%), unless specified otherwise.

b Defined as admission to the intensive care unit for any period of time.

c Total antibiotic spectrum index per patient per day, a metric designed to compare a spectrum of activity ranging from the most narrow at 1 (eg, dicloxacillin) to the broadest spectrum at 13 (eg, tigecycline).

### Microbiological characteristics

Forty-three patients (36.4%) had at least one microorganism identified through microbiological testing during evaluation for NV-HAP. The most common positive microbiological tests included lower respiratory specimens (n = 35) and nasal swabs for *S. aureus* (n = 6). Overall, the most common gram-positive organisms identified were *S. aureus* (n = 18), *Streptococcus pneumoniae* (n = 5), and *Enterococcus* species (n = 4); the most common gram-negative organisms identified included *Pseudomonas aeruginosa* (n = 5) and *Stenotrophomonas maltophilia* (n = 2).

### Antibiotic characteristics

All patients were prescribed antibiotics during hospitalization with 66.9% additionally receiving postdischarge antibiotics. The most frequently administered antibiotics during hospitalization included glycopeptides (72.9%) and penicillins (62.7%; most were combination β-lactams/β-lactamase inhibitors). Carbapenems (3.4%), lincosamides (2.5%), and aminoglycosides (0.8%) were rarely used. No patients received a monobactam, oxazolidinone, or polymyxin.

### Antibiotic duration of therapy and outcomes

Among all patients, 5% were detected with *C. difficile*, 9% were detected with a new multidrug-resistant organism, 43% were readmitted, and 33% died within 90 days of discharge. When compared with a duration of ≤7 days, prolonged durations of 8–14 days and ≥15 days were not associated with reduced adjusted odds of 90-day readmission or death after accounting for intensive care unit admission (Table 2). Intensive care unit admission was associated with increased odds of 90-day all-cause mortality (odds ratio, 2.77 [95% CI, 1.08–7.10], *P* = .03).

**Table 2.** Factors associated with all-cause readmission and mortality within 90 days of discharge in a logistic regression model

Variable	All-cause readmission		All-cause mortality	
	Odds ratio (95% CI)	*P* value	Odds ratio (95% CI)	*P* value
Antibiotic duration				
≤7 days	1.00	–	1.00	–
8–14 days	0.75 (0.28, 2.02)	0.57	0.63 (0.22, 1.83)	0.40
≥15 days	0.70 (0.21, 2.36)	0.56	1.58 (0.45, 5.55)	0.48
Intensive care unit admission^a^	1.25 (0.51, 3.07)	0.62	2.77 (1.08, 7.10)	0.03

a Defined as admission to the intensive care unit for any period of time.

## Discussion

In this cohort of older adults living with advanced cancer who developed NV-HAP following receipt of palliative chemotherapy, we found no association between the duration of antibiotic therapy and all-cause mortality or all-cause readmission within 90 days of discharge after accounting for intensive care unit admission. Our study highlights an important patient population that is often hospitalized and exposed to antibiotic therapy when symptom relief is a primary goal of care.^7^ Findings from this investigation suggest no identified benefit of prolonged antibiotic therapy for NV-HAP in older adults with advanced cancer. These results, combined with prior evidence underscoring the high potential for harm of antibiotics in older adults with terminal illness, may inform antimicrobial stewardship efforts in palliative care settings.^8^

This study builds on literature demonstrating similar outcomes between short-course and prolonged-course antibiotic therapy for hospital-acquired pneumonia.^9^ Consistent with prior studies, we found that a shorter duration of therapy is not associated with differential clinical outcomes with respect to readmission or mortality for NV-HAP among older adults with advanced cancer. In contrast, intensive care unit admission was an independent predictor of death, suggesting that the severity of an underlying disease and the need for critical care are more influential factors in determining clinical outcomes than the duration of antibiotic therapy.

In this study, over 80% of patients received >7 days of therapy for NV-HAP, a duration that is discordant with clinical guidelines.^10^ This discrepancy highlights an opportunity for antimicrobial stewardship to improve guideline adherence. Future studies may consider engaging diverse stakeholders to inform multifaceted antimicrobial stewardship strategies in older adults with advanced cancer.

This study had limitations. As a single-center investigation, external validity was limited, and outcomes from external institutions were unrecorded. Given the wide confidence intervals, the sample size was small, and the power may have been limited. Additionally, there was potential for observation bias during medical record review. Furthermore, the symptom duration, quality of life, and length of stay were not assessed and may have been associated with antibiotic exposure duration.^7^ Despite limitations, this study shows that prolonged antibiotic durations for NV-HAP were not associated with reduced odds of readmission or death among older adults with advanced cancer. These data may reinforce recommendations for shorter durations of therapy for NV-HAP in this population.
